# Supplementary material for: Genomic insights into clonal diversity in UK populations of the potato aphid, Macrosiphum euphorbiae
Source: BMC Genomics. 2025 Nov 11;26:1025. doi: 10.1186/s12864-025-12152-1 (PMC12606827; doi:10.1186/s12864-025-12152-1)
Supplement: Supplementary file 2 — Supplementary Material 2. [file 12864_2025_12152_MOESM2_ESM.docx]

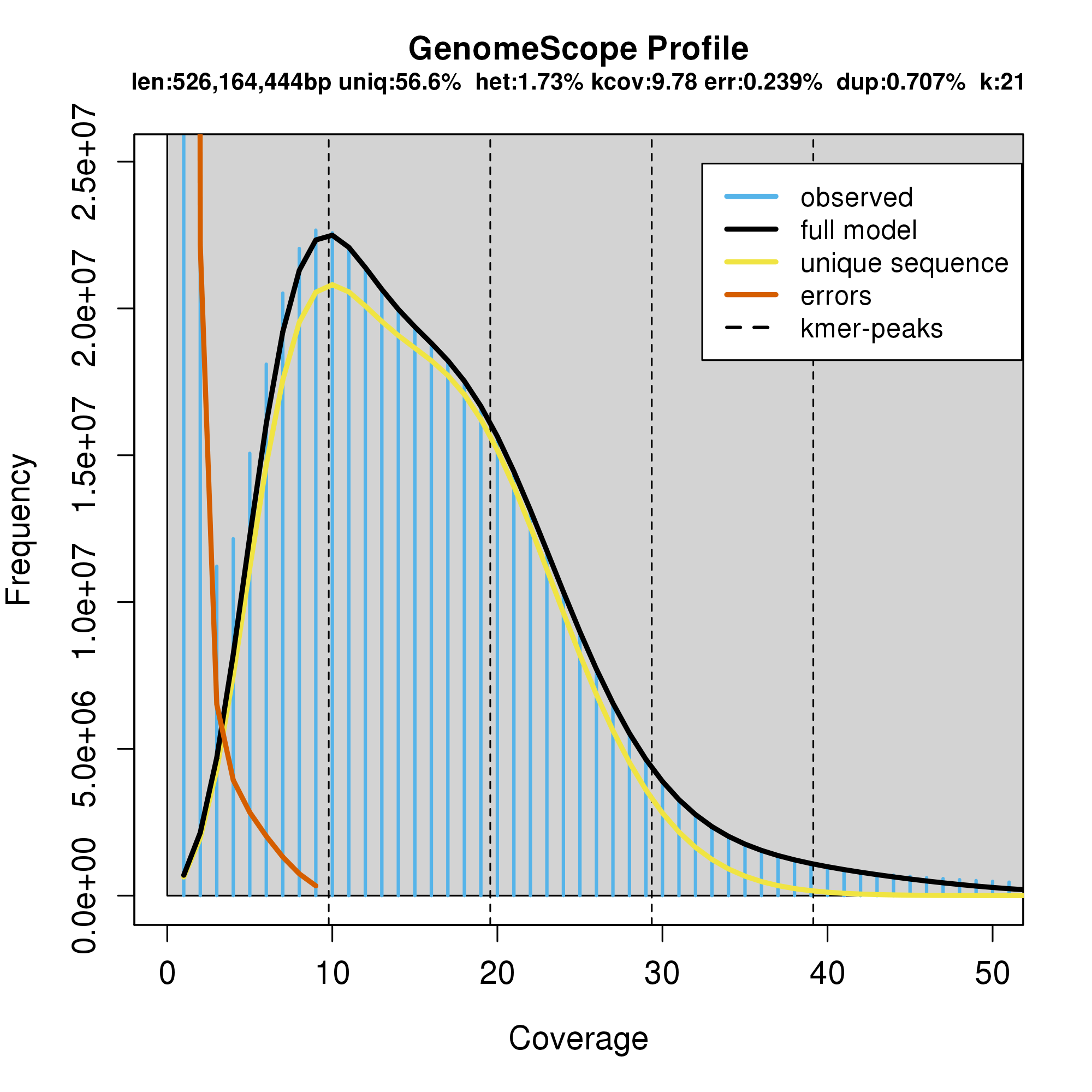


**Supplementary figure 1. K-mer analysis of Illumina short reads for the genotype 1 line MW16/67.** Genomescope predicts a genome size of 526 Mb, consistent with the predicted value in Wenger *et al.* (2017) of 530 Mb. GenomeScope also predicts more than 40% of the genome consists of repeat structures, as well divergence between haplotypes based on heterozygosity score.


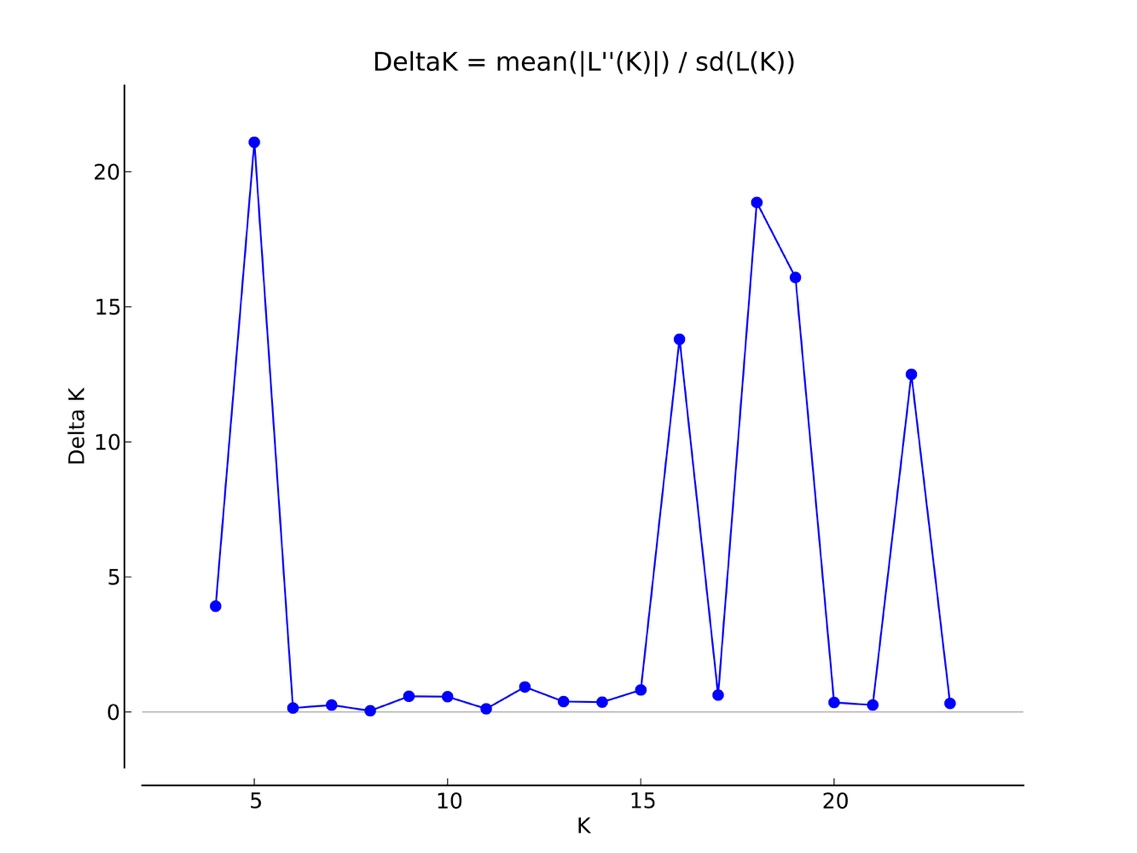


**Supplementary figure 2. Predicted value of genetic clusters (*K*) for *M. euphorbiae* based on the delta *K* method implemented in STRUCTURE HARVESTER (Earl & von Holdt, 2012).** Briefly, delta K is calculated through measuring the highest likelihood of each value of *K*, followed by assessing the variance of likelihood values between replicates within each *K.*

**
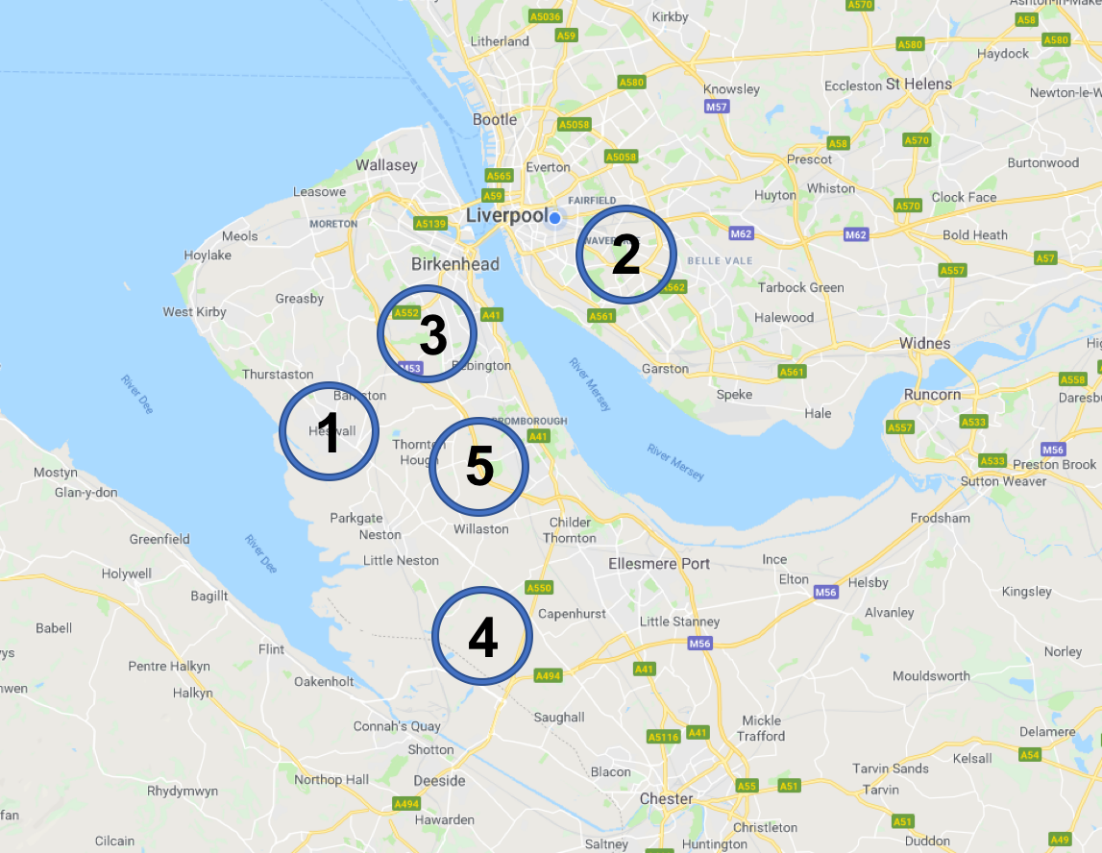
**

**Supplementary Figure 3. Locations of Merseyside sites used for aphid sampling.** Also see supplementary table 2.

**
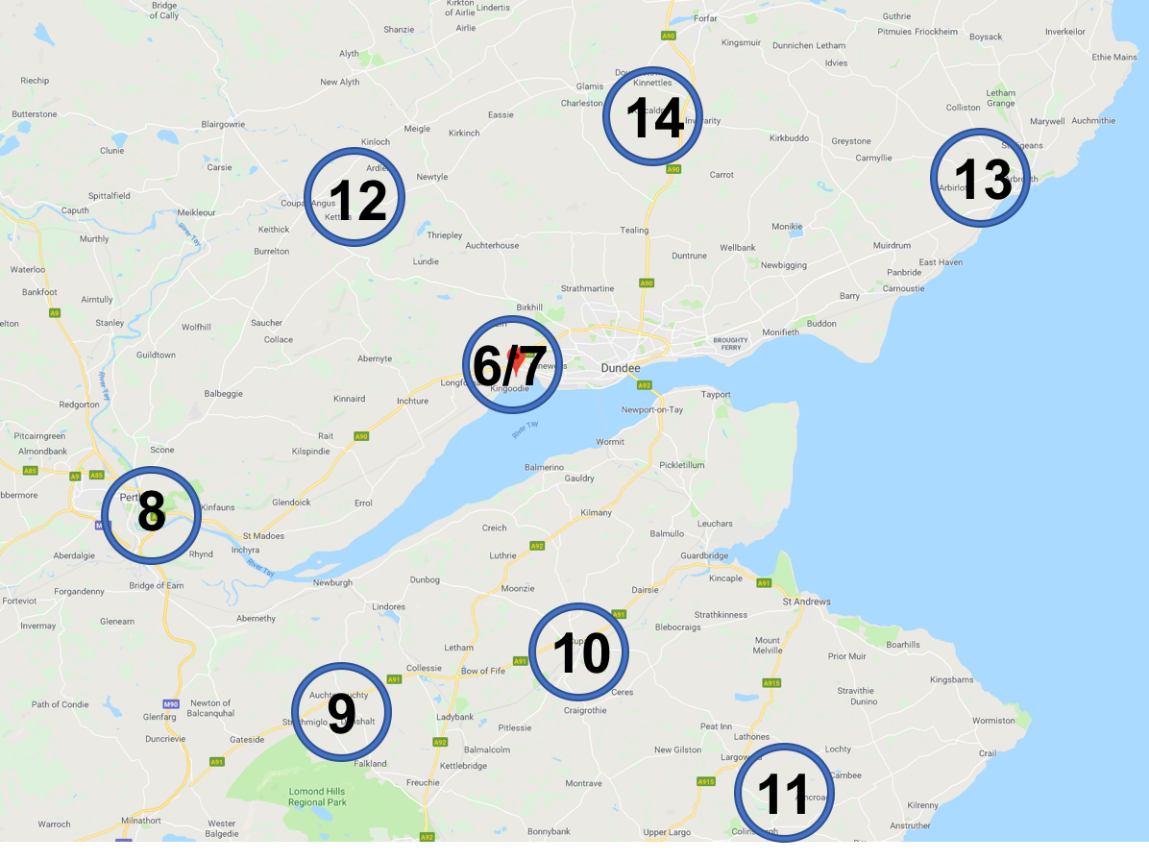
**

**Supplementary Figure 4. Locations of sites in Tayside, Perth and Fife used for aphid sampling.** Also see supplementary table 2.
