# Supplementary material for: Genomic insights into clonal diversity in UK populations of the potato aphid, Macrosiphum euphorbiae
Source: BMC Genomics. 2025 Nov 11;26:1025. doi: 10.1186/s12864-025-12152-1 (PMC12606827; doi:10.1186/s12864-025-12152-1)
Supplement: Supplementary file 4 — Supplementary Material 4. [file 12864_2025_12152_MOESM4_ESM.docx]

**Description of additional data files**

Additional file 1 **- supplementary_tables_data_sources.xlsx**

- Title: Supplementary Tables
- Description: Collation of all supplementary tables referred to in the main text

Additional file 2 - **supplementary_figures.docx**

- Title: Supplementary figures
- Description: Collation of all supplementary figures referred to in the main text

Additional file 3 **- supplementary_methods.docx**

- Title: Supplementary methods
- Description: Methods for high molecular weight DNA extraction and genome assessment tool Merqury.
